# P-1889. Penicillin Allergy and Its Impact on Clinical Outcomes in OPAT Patients: A Retrospective Cohort Study

**DOI:** 10.1093/ofid/ofae631.2050

**Published:** 2025-01-29

**Authors:** Maher Jafar, Kristen McSweeney, Fang-yu Liu, Rachel Erdil, Tine Vindenes, Shira Doron, Kap Sum Foong

**Affiliations:** Tufts Medical Center, Boston, Massachusetts; Tufts Medical Center, Boston, Massachusetts; Tufts Medical Center, Boston, Massachusetts; Tufts Medical Center, Boston, Massachusetts; Tufts Medical Center, Boston, Massachusetts; Tufts Medical Center, Boston, Massachusetts; Tuft Medical Center, Boston, MA

## Abstract

**Background:**

10% of the U.S. population is labeled as allergic to penicillin, with higher rates among women and older adults. These individuals often experience worse clinical outcomes. Despite the extensive use of Outpatient Parenteral Antimicrobial Therapy (OPAT), data on the prevalence of penicillin allergy in this population and its impact on clinical outcomes are scarce. Our study aims to address this knowledge gap by assessing rates of penicillin allergy in OPAT patients and its associations with OPAT-related clinical outcomes.

Table 1

**Figure:**
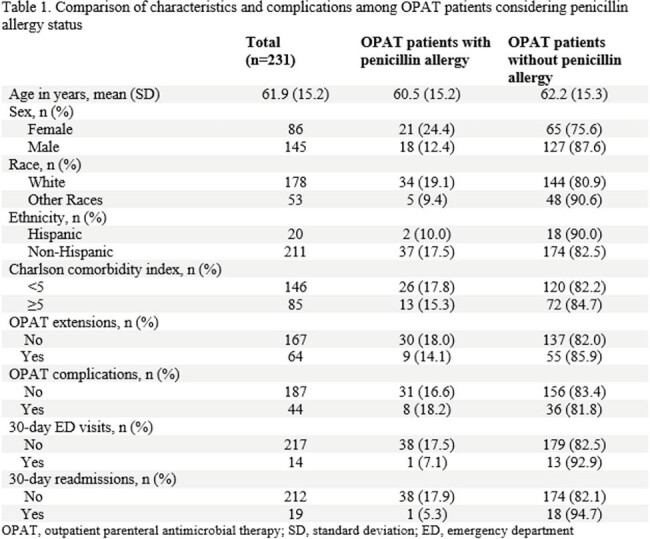

Comparison of characteristics and complications among OPAT patients considering penicillin allergy status

**Methods:**

We conducted a retrospective cohort study at Tufts Medical Center, including all patients aged ≥18 years discharged on OPAT between April 2022 and October 2022. Data on demographics, penicillin allergy status, complications, and outcomes were extracted through chart reviews. The primary outcomes were: (1) rates of penicillin allergy, and (2) association between penicillin allergy and OPAT complication rate (line- or antibiotic-related), OPAT extension, 30-day ED visits, and readmission rates. We used a multivariable logistic regression model to examine these associations, adjusting for age, sex, race, ethnicity, and Charlson comorbidity index. Two-sided p values were considered statistically significant at .05.

Table 2

**Figure:**
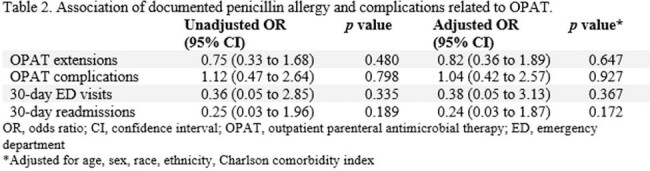

Association of documented penicillin allergy and complications related to OPAT

**Results:**

A total of 231 patients received OPAT; 39 (17.9%) had documented penicillin allergy. Females and white patients had higher rates of penicillin allergy compared to their counterparts; 24.4% vs 12.4% and 19.1% vs 9.4%, respectively (Table 1). In the final multivariable logistic regression model, penicillin allergy status was not associated with OPAT extension (adjusted odds ratios[aOR] 0.83; 95% confidence interval[CI] 0.36-1.89), OPAT-related complications (aOR 1.04; 95%CI 0.42-2.57), 30-day ED visits (aOR 0.38, 95%CI 0.05-3.13), and 30-day readmissions (aOR 0.24; 95%CI 0.03-1.87) (Table 2).

**Conclusion:**

Our findings suggest that the rates of PCN allergy were higher than in hospital settings (∼15%), but these do not significantly influence the OPAT clinical outcomes. However, neither group met the required 248 subjects to reliably detect a 10% increase in complication rates, limiting the significance of the findings. Further research with a larger sample size is needed to validate these findings.

**Disclosures:**

Shira Doron, MD, Medtronic: Expert Testimony|Sunovion: Advisor/Consultant|Vertex: Speaker

